# The effect of walnut intake on factors related to prostate and vascular health in older men

**DOI:** 10.1186/1475-2891-7-13

**Published:** 2008-05-02

**Authors:** Kim J Spaccarotella, Penny M Kris-Etherton, William L Stone, Deborah M Bagshaw, Valerie K Fishell, Sheila G West, Frank R Lawrence, Terryl J Hartman

**Affiliations:** 1 Department of Nutritional Sciences, Rutgers University, New Brunswick, NJ 08901, USA; 2 Department of Nutritional Sciences, The Pennsylvania State University, University Park, PA 16802, USA; 3 Department of Pediatrics, East Tennessee State University, Johnson City, TN 37614, USA; 4 Department of Biobehavioral Health, The Pennsylvania State University, University Park, PA 16802, USA; 5 Department of Human Development and Family Studies, The Pennsylvania State University, University Park, PA 16802, USA

## Abstract

**Background:**

Tocopherols may protect against prostate cancer and cardiovascular disease (CVD).

**Methods:**

We assessed the effect of walnuts, which are rich in tocopherols, on markers of prostate and vascular health in men at risk for prostate cancer. We conducted an 8-week walnut supplement study to examine effects of walnuts on serum tocopherols and prostate specific antigen (PSA). Subjects (*n *= 21) consumed (in random order) their usual diet +/- a walnut supplement (75 g/d) that was isocalorically incorporated in their habitual diets. Prior to the supplement study, 5 fasted subjects participated in an acute timecourse experiment and had blood taken at baseline and 1, 2, 4, and 8 h after consuming walnuts (75 g).

**Results:**

During the timecourse experiment, triglycerides peaked at 4 h, and gamma-tocopherol (γ-T) increased from 4 to 8 h. Triglyceride – normalized γ-T was two-fold higher (*P *= 0.01) after 8 versus 4 h. In the supplement study, change from baseline was +0.83 ± 0.52 μmol/L for γ-T, -2.65 ± 1.30 μmol/L for alpha-tocopherol (α-T) and -3.49 ± 1.99 for the tocopherol ratio (α-T: γ-T). A linear mixed model showed that, although PSA did not change, the ratio of free PSA:total PSA increased and approached significance (P = 0.07). The α-T: γ-T ratio decreased significantly (*P *= 0.01), partly reflecting an increase in serum γ-T, which approached significance (*P *= 0.08).

**Conclusion:**

The significant decrease in the α-T: γ-T ratio with an increase in serum γ-T and a trend towards an increase in the ratio of free PSA:total PSA following the 8-week supplement study suggest that walnuts may improve biomarkers of prostate and vascular status.

## Introduction

Cancer is a major cause of mortality worldwide. In the United States, it is second only to heart disease [1]. Prostate cancer is the most common type of cancer among men, with an incidence rate of one in six men [2]. Furthermore, because androgens may play a role in the smooth muscle proliferation associated with the development of both prostate cancer and CVD, men at increased risk for prostate cancer also may be at increased risk for heart disease [3]. As many as one in four men over 60 years old have symptoms of both prostate enlargement and high blood pressure [3] and therefore have an increased risk for both diseases [4]. Moreover, although a PSA concentration of 4.0 ng/mL (4.0 μg/L) or greater is often used in screening for prostate cancer, more than 20% of men with diagnosed prostate cancer have PSA levels below this cut-point [5]. Thus, recent work has focused on using percent free PSA (PFP) in combination with total PSA to improve specificity of diagnosis and decrease false-positives [5]. Since prostate cancer and heart disease affect many men, there is a need for affordable and practical preventive options that target both diseases.

Epidemiologic studies suggest that the antioxidant vitamin E, particularly the γ-T form, may hold promise in decreasing risk of prostate cancer [6-8] and CVD [9-11]. In cell culture studies, both α-T and γ-T inhibit proliferation of vascular smooth muscle cells [12,13], an important event in atherogenesis [12]. Furthermore, both α-T and γ-T inhibit growth of malignant cells, including prostate tumor cells [14-16], and a recent supplementation study reported that the combination of α-T and γ-T significantly increased nitric oxide release and was more potent in preventing platelet aggregation than was α-T alone [17,18].

Walnuts may provide an inexpensive and practical method for supplementing intake of both tocopherols and other nutrients that may protect against prostate cancer. For example, 75 g of walnuts contain 0.52 mg α-T and 15.6 mg γ-T [19]. Walnuts also contain ellagic acid (590 μg/g) [20], which has been shown to effectively induce apoptosis and inhibit angiogenesis [21,22]. In addition, walnuts are a rich source of unsaturated fatty acids that favorably affect CVD risk [23], and several recent feeding studies with walnuts have reported a total and LDL cholesterol lowering effect following consumption of about 70–80 g/d of walnuts [24-26].

The primary goal of the supplementation study was to evaluate the effects of consuming 75 g/d of walnuts on metabolic factors related to prostate cancer and CVD in men at risk for these diseases. Furthermore, in order to develop interventions using dietary γ-T as a preventive strategy in prostate cancer and CVD, it is important to understand its bioavailability. Previous work has shown a significant increase in plasma γ-T following a fat-rich meal supplemented with 0.4–0.8 mg γ-T per kilogram of body weight [27], and walnuts contain polyunsaturated fatty acids, which may protect against CVD [23] and may enhance tocopherol absorption [28]. Our results indicate that γ-T in walnuts is absorbed, demonstrating bioavailability from walnuts, which provided the requisite assurance needed to conduct a longer term study to evaluate the effects of γ-T on prostate and vascular health.

## Methods

### Subjects

Participants were healthy, non-smoking men between 45 and 75 years of age who resided in central Pennsylvania. Participants had total PSA ≥ 2.0 ng/mL (2.0 μg/L), but they did not have clinically diagnosed prostate cancer. Newspaper ads and fliers were used for recruitment, and potential subjects were screened by telephone. Exclusion criteria included allergies to nuts and use of prescription and non-prescription preparations known to alter PSA (e.g. Saw Palmetto, Finesteride), hormone levels, blood pressure or blood lipids. Men taking vitamin E supplements were eligible if they discontinued use two months before entering the study. Those who were eligible were invited to a screening visit during which blood pressure and PSA level were measured. Written informed consent was obtained from each participant, and the Human Subjects Committee at the Pennsylvania State University approved the study.

#### Acute timecourse experiment

##### Study design

To verify that γ-T in the walnuts was absorbed, we conducted an acute timecourse experiment prior to the feeding study at the General Clinical Research Center (GCRC) in University Park, PA. Participants (*n *= 5) were part of a controlled feeding study in which walnuts accounted for 24% of total daily calories and had been following their diet for 3 weeks to allow them to acclimate to the diet and ensure stable baseline measures [29].

Following an overnight fast and a baseline blood collection, they consumed 75 g of walnuts. Postprandial changes in serum concentrations of triglycerides, cholesterol and tocopherols were assessed at 1, 2, 4 and 8 hours after the walnuts were consumed, similar to acute timecourse experiments with serum lipids, which typically measure concentrations over an 8-hour period [30-32].

##### Biochemical analyses

The Pediatric Research Laboratory at East Tennessee State University analyzed serum triglycerides, total cholesterol and tocopherols. The tocopherols were extracted with ethanol:hexane and analyzed using a modification of the HPLC method described by Bieri [33]. An aliquot of plasma incubation mixture was mixed with ethanol containing tocol as an internal standard and immediately extracted into hexane containing BHT (227 μmol/L). The hexane layer was removed and evaporated under a gentle stream of nitrogen gas. The residue was dissolved in ethanol containing propyl gallate (236 mol/L) as an antioxidant and injected into a reverse phase Altex-Ultrasphere-ODS (Beckman Instruments, Inc., Fullerton, CA) 4.6 mm ID × 25 cm column. The mobile phase was methanol:water (99.5%:0.5%, vol:vol). A McPherson Model FL-750 fluorescence detector (McPherson Instruments, Acton, MA) was utilized with 294 nm excitation and 324 nm emission.

Enzyme kits from STANBIO (Boerne, TX) were used to assay total cholesterol and triglycerides. Intra-assay coefficients of variation were 2.5% for total cholesterol and 3% for triglycerides. Batches of subject samples were assayed with each subject's samples grouped together in random order.

##### Statistical methods

All statistical analyses were performed in SAS, version 8.0 (SAS Institute, Cary, NC), and differences were considered significant at *P *< 0.05. All values are least-squares means ± SEM. Statistical analyses were performed using a linear mixed model with time as the repeated factor.

#### Walnut supplement study

##### Study design

The study was designed as a two-period cross-over in which participants received two treatments in random order. Participants (*n *= 21) spent the first eight-week period on either a walnut supplement diet, in which they consumed 75 g of walnuts per day (approximately 24% of energy intake from walnuts, based on a diet with 2000 kcal.day), or their usual diet ("Average American Diet"); then, following a two-week break, they switched to the other diet for eight-weeks. Participants kept five, three-day food records, one set at baseline and additional records at weeks 4 and 8 of each diet period. Clinic visits took place at the GCRCs located in University Park, PA and Hershey, PA (at the Milton Hershey Medical Center). Endpoint clinic visits included blood pressure, weight measurement and fasting blood collection (0.05 L). Participants were instructed to maintain their baseline weight and activity level throughout the study. To ensure that weight remained constant during the walnut supplement diet, subjects were instructed on strategies for isocalorically substituting walnuts for other energy sources in the diet. Participants were weighed while wearing indoor clothing but without shoes on digital scales at weeks 1, 2, 3, 4, 6 and 8. During weeks 4 and 8 of both diet periods, participants met with a registered dietitian, who reviewed the food records with participants to reinforce compliance and suggest individualized strategies for weight maintenance.

##### Diet

During the walnut supplement diet, participants received 75 g of walnuts per day. The walnuts were shelled and unroasted English walnuts purchased from a single lot, pre-weighed and packaged in 75 g amounts and stored frozen at -17°C. Participants were instructed to replace another fat source in their usual diet with the walnuts and to consume the walnuts throughout the day with snacks and meals to maximize tocopherol absorption [28,34].

Food record data were analyzed for intakes of energy, fat, tocopherols and other nutrients using Nutrition Data System for Research software, version 5.0_35, 2004 (Nutrition Coordinating Center, University of Minnesota, Minneapolis, MN.)

##### Urinary symptoms

At endpoint clinic visits, participants completed a nine-item American Urological Association questionnaire measuring urinary symptoms [35]. The first seven questions asked participants to indicate, from 0 (not at all or none) to 5 (almost always or ≥ 5 times per night), their frequency of specific urinary symptoms, such as a need to urinate within two hours of finishing urination or difficulty postponing or beginning urination, in the last month. Participants then totaled these scores to obtain a "symptom score." The questionnaire was scored as follows: a "total symptom score" of 1–7 indicated that urinary symptoms were mild (1 being the mildest); 8–19 indicated that symptoms were moderate, and a score of 20–35 indicated that symptoms were severe (35 being the most severe).

##### Biochemical Endpoints

The sample analysis focused on serum biomarkers and hormones that have been consistently associated with prostate disease and CVD in past studies. The analyses measured total, free and percent free PSA, testosterone (total, bioavailable and percent bioavailable), 3α-androstanediol glucuronide (a metabolite of dihyrotestosterone), estradiol, IGF-1, total cholesterol and lipids, oxidized LDL and α- and γ-T. At endpoint clinic visits, twelve-hour fasting blood samples were collected into Vacutainer tubes (for the tocopherol analysis, samples were drawn into Vacutainer tubes containing EDTA). Following collection, samples were centrifuged at 2600 × g for 15 min. Plasma for the tocopherol analyses was treated with propyl gallate, an antioxidant preservative, and all samples were frozen at -70°C until analysis at the end of the study.

The GCRC Core Laboratory at Hershey Medical Center conducted the assays for serum hormones, IGF-1 and PSA levels, and the Pediatric Research Laboratory at East Tennessee State University analyzed the triglycerides, total cholesterol and tocopherols as described above for the acute timecourse experiment. Total and free PSA levels were determined using microparticle enzyme immunoassay on an automated immunoassay platform (Abbott AxSYM, Abbott Laboratories, Chicago, Illinois). Total testosterone and estradiol were measured using solid-phase radioimmunoassays. 3α-androstanediol glucuronide was determined using a competitive, double antibody radioimmunoassy with rabbit anti-androstanediol glucuronide. IGF-1 was determined by chemiluminescence using the Nichols Advantage automated system (Nichols Institute Diagnostics, Inc., San Juan Capistrano, CA). Free and weakly-bound testosterone were measured using ammonium sulfate precipitation of sex-hormone binding globulin-bound testosterone following equilibration of 3H-testosterone with the binding proteins in the serum sample. Percent free testosterone was determined by scintillation counting of the supernatants.

Enzyme kits from STANBIO (Boerne, TX) were used to assay HDL cholesterol, and the intra-assay coefficient of variation was 2.2%. LDL cholesterol was calculated using the Friedewald equation [36], and oxidized LDL was measured using an enzyme kit from Alpco Diagnostics (Windham, NH) with an intra-assay coefficient of variation of 3.6%. The laboratories analyzed batched samples of blood with each subject's samples grouped together in random order.

##### Blood Pressure Measurement

Blood pressure was measured by registered nurses using a sphygmomanometer. Measurements were taken from the right arm and were made after a 5-minute rest in the sitting position with both feet flat on the floor. Two measurements were made at each visit and averaged.

##### Statistical methods

All outcome variables were tested for sequence and period effects using a linear mixed model; none were found, and these factors were dropped from the final models. A paired sample t-test (two-sided) was used to compare participants' responses to the urinary symptoms questionnaire's "total symptom" score and to compare blood pressure and dietary intake of energy, total fat and vitamin E between the two diets. To measure change in lipids, PSA, vitamin E and hormones, a difference score (Δ) between serum concentrations of each variable at baseline and each diet period was calculated. For example, for each variable, Δ_a _= concentration following usual diet – concentration at baseline and Δ_w _= concentration following intervention diet – concentration at baseline. Because body weight change differed between the two diets (see below), we tested whether covarying for change in weight (vs. screening) significantly impacted diet-related change in each of the outcome variables. Based on these analyses, change in weight was included in the analyses of tocopherols and IGF-1. In addition, fasting TG and PFP concentrations were not normally distributed, so log-transformed (base 10) concentrations were used in the mixed model analyses and the resultant change scores and means reported as geometric means. Values are reported as least-squares means ± SEM unless otherwise noted.

## Results

### Acute timecourse experiment

**Figure 1:**
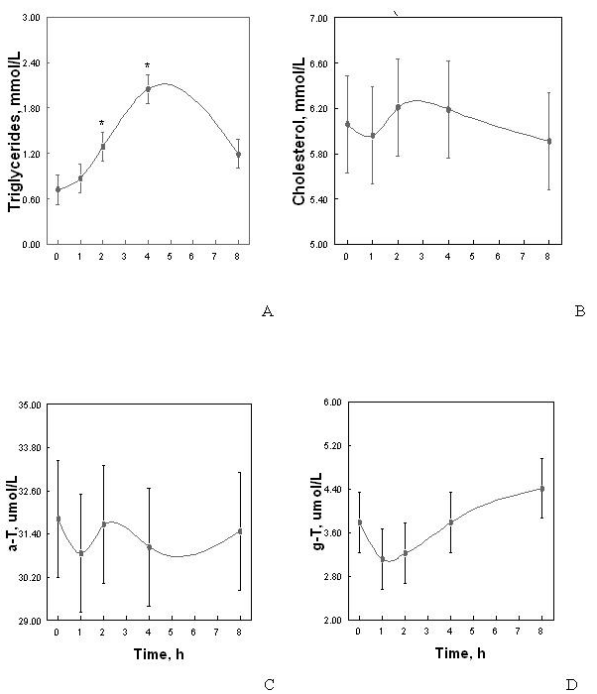
Effect of a 75 g walnut bolus on average serum concentrations of triglycerides (A), cholesterol (B), α-tocopherol (a-T) (C) and γ-tocopherol (g-T) (D) over 8 h timecourse following consumption (time 0). Values are least-squares means ± SEM, n = 5. Asterisks indicate *P equal to or less than 0.05.

Following consumption of 75 g of walnuts, the average concentration of serum triglycerides rose from 0.87 ± 0.19 mmol/L at 1 h to a peak of 2.05 ± 0.19 mmol/L at 4 h. The average concentration of serum total cholesterol was 5.95 ± 0.43 mmol/L at 1 h and peaked at 6.21 ± 0.43 mmol/L 2 h after walnut consumption (Figure 1). Average serum γ-T reached peak concentration of 4.41 ± 0.55 μmol/L between 4 and 8 h (Figure 1). Average serum α-T reached peak concentration of 32.4 ± 1.63 μmol/L between 2 and 4 h (Figure 1). Compared to baseline, serum triglycerides were significantly elevated at 2 and 4 h (mean concentrations of 1.29 ± 0.19 mmol/L, *P *= 0.05 at 2 h and 2.05 ± 0.19 mmol/L, *P *< 0.0001 at 4 h) (Figure 1). In the final model, changes in total cholesterol and tocopherols were not significant over time (*P *= 0.98 for cholesterol, *P *= 0.98 for α-T and *P *= 0.49 for γ-T), and changes in tocopherol were not significant when adjusted for cholesterol. However, we observed a significant, two-fold increase (*P *= 0.01) in the ratio of γ-T to serum triglycerides at 4 h compared to 8 h.

### Walnut supplement study

#### Subject characteristics

Twenty-two white males from central Pennsylvania volunteered to participate in the walnut supplement study; one participant with a prior history of gastrointestinal distress withdrew following a reoccurrence of symptoms during the walnut diet. The average age was 65.9 years (range was 55 to 75 years). Mean weight was 84.8 ± 2.9 kg at the screening visit, 83.4 ± 2.8 kg at the end of the usual diet and 84.3 ± 2.9 kg at the end of the walnut supplement diet. Mean body mass index was 27.5 ± 3.5 during the usual diet and 27.8 ± 3.7 during the walnut diet. Subjects on the usual diet lost 1.36 ± 0.39 kg (*P *= 0.001), and subjects on the walnut supplement diet maintained their weight. Importantly, there was no weight gain during the walnut supplement diet period.

#### Dietary intake

Aside from the nutrients provided by the walnuts, the participants' diets were similar in energy, total fat and α-T during both diet periods (Table 1). The 75 g of walnuts consumed each day during the walnut supplement diet contained 490 kcal of energy, 48.9 g of fat, 0.52 mg of α-T and 15.6 mg of γ-T [19], and a nutrient analysis of a sample of the walnuts used in our study showed that they contained about 23 mg of γ-T and trace amounts of α-T (data not shown), as expected. Analysis of the participants' diet records during the walnut supplement diet period showed significant increases (values are means ± SD) in energy (478 ± 616 kcal/d; *P *= 0.003), total fat (44.4 ± 30.9 g/d; *P *< 0.0001), monounsaturated fat (6.9 ± 13.3 g/d; *P *= 0.02), polyunsaturated fat (29.1 ± 18.3; *P *< 0.0001) and γ-T (13.7 ± 7.99 mg/d; *P *< 0.0001), all of which were consistent with the amount of each of these nutrients present in 75 g of walnuts (Table 1), suggesting that participants were compliant with the walnut treatment.

**Table 1:** Average intake from food records collected during feeding study – Average American vs. Intervention Diet^1^

	Average American Diet	Intervention Diet, intake with walnuts	*P*^2^
Total energy, *kcal/d*	2041 ± 504	2519 ± 512	0.003
Total fat, *g/d*	81.1 ± 24.6	125 ± 21.2	< 0.0001
Saturated fat, *g/d*	29.7 ± 9.86	32.2 ± 9.04	0.28
Monounsaturated fat, *g/d*	30.4 ± 9.74	37.3 ± 7.79	0.02
Polyunsaturated fat, *g/d*	16.2 ± 10.1	45.2 ± 11.2	< 0.0001
Alpha-tocopherol, *mg/d*	9.32 ± 6.50	14.1 ± 8.01	0.63
Gamma-tocopherol, *mg/d*	15.0 ± 5.93	28.6 ± 7.81	< 0.0001

^1^Values are means ± SD, *n *= 20.

^2^*P *for comparison of Average American Diet and Intervention Diet With Walnuts.

#### Serum biomarkers and blood pressure

After controlling for weight, no significant differences were detected in serum α-T, although the change in serum γ-T approached significance (Table 2). However, after the walnut supplement diet, the ratio of serum α-T:γ-T decreased significantly (Δ_a _= -0.51 ± 1.99, Δ_w _= -3.49 ± 1.99 μmol/L; *P *= 0.01) (Table 2). There were no significant treatment effects on serum concentrations of triglycerides, total cholesterol, LDL cholesterol, bioavailable testosterone, total PSA, free PSA, PFP or weight-adjusted IGF-1 (Table 2), or on systolic blood pressure (Δ_a _= -1.65 ± 2.78, Δ_w _= -3.05 ± 2.78 mmHg; *P *= 0.61) and diastolic blood pressure (Δ_a _= -2.50 ± 1.52 mmHg, Δ_w _= -3.45 ± 1.52 *P *= 0.54). However, a linear mixed model showed that, although PSA did not change, the ratio of free PSA:total PSA increased and approached significance (P = 0.07).

**Table 2:** Mean change from baseline in serum biomarkers during feeding study – Average American vs. Intervention Diet^1^

	Baseline	Change Following Average American Diet^2^	Change Following Intervention Diet^2^	P-value for Difference in Change
Total cholesterol, *mmol/L*	5.01 ± 0.16	-0.02 ± 0.12	-0.18 ± 0.12	0.13
HDL, *mmol/L*	1.36 ± 0.06	-0.04 ± 0.04	-0.05 ± 0.04	0.95
Oxidized LDL, *mmol/L*	2.61 ± 0.16	-0.15 ± 0.11	-0.16 ± 0.11	0.98
Triglycerides^3^, *mmol/L*	1.31 ± 1.01	1.00 ± 1.00	1.00 ± 1.00	0.48
LDL, *mmol/L*	3.11 ± 0.15	0.03 ± 0.13	-0.10 ± 0.13	0.21
LDL:HDL	2.37 ± 0.16	0.15 ± 0.15	0.04 ± 0.15	0.34
Non-HDL:HDL	2.79 ± 0.19	0.18 ± 0.15	0.03 ± 0.15	0.28
Total PSA, μ*g/L*	5.02 ± 0.63	-0.07 ± 0.35	0.05 ± 0.36	0.75
Free PSA, μ*g/L*	0.92 ± 0.13	0.05 ± 0.10	0.13 ± 0.10	0.56
Percent Free PSA^3^, *%*	38.8 ± 1.04	1.03 ± 1.03	0.99 ± 1.03	0.07
Testosterone *nmol/L*	16.4 ± 1.11	-0.60 ± 0.70	-0.75 ± 0.70	0.82
Bioavailable testosterone *nmol/L*	4.66 ± 0.29	-0.35 ± 0.26	-0.34 ± 0.26	0.99
Percent bioavailable testosterone, *%*	0.92 ± 0.13	-0.85 ± 0.93	-0.45 ± 0.94	0.66
IGF-1, *μg/L*	143 ± 9.11	10.6 ± 5.85	1.79 ± 6.07	0.13
3α-Androstanediol glucuronide, *nmol/L*	13.8 ± 1.55	-0.02 ± 0.01	-0.02 ± 0.01	0.90
Estradiol, *pmol/L*	97.7 ± 5.74	1.22 ± 4.02	-3.85 ± 4.02	0.34
Alpha-tocopherol,^4 ^*μmol/L*	29.3 ± 2.19	-0.71 ± 1.30	-2.65 ± 1.30	0.13
Gamma- tocopherol,^4 ^*μmol/L*	4.14 ± 0.43	0.26 ± 0.52	0.83 ± 0.52	0.08
Tocopherol ratio alpha:gamma^4^	11.0 ± 9.91	-0.51 ± 1.99	-3.49 ± 1.99	0.01

^1^Values are least-squares means ± SEM, *n *= 21. Data presented from the final model, in which weight was significant only for tocopherols and IGF-1 and was omitted for all other variables.

^2^Change scores calculated for each diet as Δ = mean concentration following diet – mean concentration at baseline.

^3^Triglyceride and percent free PSA (*n *= 20) change score data were log-transformed, and change scores are presented as geometric means.

^4 ^*n *= 20. Tocopherol change score adjusted for change in weight between the two diets.

#### Urinary symptoms

The average score was 7.33 ± 5.83 following the usual diet and 6.95 ± 5.66 following the walnut supplement diet. The "total symptom score" was similar for both diets (*P *= 0.49).

## Discussion

The present study showed that substituting 3 oz of walnuts per day for fat sources in an Average American Diet significantly increased intake of γ-T and mono- and polyunsaturated fats and resulted in a significant decrease in serum α-T:γ-T, with a trend towards an increase in serum γ-T and PFP, in this population of older men at risk for prostate cancer. The ratio of serum α-T:γ-T, which decreased almost four-fold, is of particular interest because it specifically contrasts changes in the two forms of vitamin E and also because it should be less affected by confounding variables such as lipids [37]. These results are consistent with a study by Lemcke-Norojarvi et al [38], which reported that feeding oils containing differing amounts of α- and γ-T resulted in an increase in serum γ-T but not α-T, and a decrease in α-T:γ-T. A previous study identified a higher ratio of serum α-T:γ-T as an important difference between patients with heart disease and healthy controls [39]. Thus, a lower ratio of serum α-T:γ-T may be protective against heart disease, and walnuts may be beneficial in lowering this ratio. Further research is needed to explore the effects of mixed tocopherols on markers of heart disease risk, such as platelet function. A recent study conducted in subjects with type II diabetes showed that, although supplementation with mixed tocopherols (500 mg/d total: 315 mg of γ-T, 75 mg of α-T and 110 mg δ-tocopherol) increased both serum α-T and γ-T and erythrocyte and platelet γ-T four-fold, it did not improve platelet activation and endothelial function [40]. The lack of an increase in α-T and the trend for a decrease in γ-T in the present study may be explained by the fact that we fed a much lower "dose" of tocopherols as walnuts in different proportions (i.e. 0.52 mg/d of α-T and 15.6 mg/d of γ-T) than did Clarke et al [40].

Our timecourse experiment confirmed that tocopherol absorption from walnuts, a whole food source of vitamin E, is similar to that which has been reported following a vitamin E supplement [27,41]. Our experiment also showed a significant increase in the ratio of γ-T to triglycerides. Although the average concentration of serum triglycerides decreased between 4 and 8 h, serum γ-T increased between 1 and 8 h (Figure 1), suggesting that the two do not move in parallel. While the action of lipoprotein lipase and the hepatic uptake of chylomicron remnants causes serum triglyceride concentrations to drop, transfer of γ-T to longer-lived lipoprotein particles may cause serum γ-T to rise over a longer period and may explain the significant increase seen in triglyceride – normalized serum γ-T. Further work is needed to determine if serum γ-T would have continued to rise beyond 8 h in our experiment. Although 8 h is sufficient for measuring maximal postprandial changes in serum lipids, our data suggest that a period of at least 8 h, and possibly longer, is needed for a maximal increase in postprandial serum γ-T from a whole food source, as has been demonstrated previously in vitamin E supplementation studies [42]. Nonetheless, our timecourse experiment does show an increase in serum γ-T over an 8 h time period indicating its bioavailability in the walnut supplement study.

Previous research suggests that walnut consumption is not associated with a higher body weight unless, as would be expected, energy intake exceeds energy expenditure [43,44]. In our study, mean weight decreased only during the usual diet period (net difference in weight change was -0.85 ± 0.37 kg between the two diets). Reported caloric intake was significantly less on the usual diet than during the walnut supplement diet, which likely explains the very modest weight loss observed. During the walnut supplement diet, even though subjects reported a higher energy intake, they maintained their body weight. Further, adjusting our results for weight as described above showed the changes in tocopherols persisted even following weight adjustment.

For several reasons, we suspect that the relative good health of our participants (aside from their prostate symptoms) may have contributed to the lack of significant treatment effects for many of our serum hormone and lipid variables. Our participants learned about the study through a newspaper advertisement targeting men concerned about elevated PSA levels and interested in using a dietary intervention to possibly improve prostate health. Consequently, they were "self-selected" and might have been more health-conscious, and thus healthier, than males of the same age in the general population. Although they had elevated PSA levels, their IGF-1 and total cholesterol levels were lower and their HDL cholesterol was higher than observed in other studies [7,45], indicating that they might have been healthier than their peers.

First, the men in our study were in the lowest quartile of serum IGF-1 when compared to individuals in the Physician's Health Study, another study with participants who were more likely to be health-conscious [7]. However, the effect size was small to moderate (0.43) for our serum IGF-1 data, indicating that a significant decrease in IGF-1 might have been detected if the sample size had been larger.

Second, among our participants, mean serum total cholesterol was lower (5.00 mmol/L, usual diet and 4.82 mmol/L, walnut supplement diet) and mean serum HDL cholesterol was higher (approximately 1.32 mmol/L, both diets) when compared to cholesterol levels of men in a large heart-health study (5.33 mmol/L, total cholesterol and 1.11 mmol/L, HDL cholesterol) [45]. Many of the previous feeding studies with walnuts that have reported favorable improvements in serum lipids have studied subjects with hypercholesterolemia [24,26,46]. Further, whereas these previous studies employed a controlled feeding design, in which participants followed a prescribed, low-fat diet, the present study employed a free-living design, in which participants followed their typical diet with or without walnuts, depending on the diet period. By nature of the design, a free-living feeding study cannot minimize dietary variation as well as a controlled feeding study, and this can make producing significant dietary change more difficult, especially given the small sample studied.

Previous research on the role of dietary γ-T in prostate cancer and CVD is limited [47]. To our knowledge, this is one of the first studies to assess the effects of walnut intake on markers of prostate health, to demonstrate the bioavailability of γ-T following walnut consumption and to contrast the postprandial timecourse of dietary lipids and tocopherols. Although the tocopherol ratio significantly improved with walnut consumption, the primary markers of prostate health (PSA and urinary symptoms) were not changed significantly by this 8-week dietary intervention. However, there was a nonsignificant increase in PFP (the ratio of free PSA: total PSA) during both diets, and research has indicated that the use of PFP in combination with total PSA may improve specificity of diagnosis and decrease false-positives [5]. Future work is needed to determine whether dietary tocopherols must be administered over longer periods of time to have a meaningful effect on prostate health and if they are effective both as a preventative measure against the development of prostate disease and as a secondary prevention strategy.

Nevertheless, the significant decrease in α-T: γ-T (without changes in body weight) in response to incorporation of 75 g of walnut/d in the diet offers the promise of significant benefits for both vascular and prostate health.

Our results are also important in light of recent research on the relationship between α-linolenic acid and prostate cancer, since walnuts are a rich source of α-linolenic acid (6.81 g/75 g walnuts) [19]. A meta-analysis of nine observational studies (four prospective studies and five nonprospective studies) assessed the relationship between prostate cancer incidence or prevalence and intake or blood levels of α-linolenic acid [48]. This meta-analysis reported that α-linolenic acid increased risk of prostate cancer (1.70; 95% CI 1.12–2.58). For the prospective studies, however, the combined estimate of relative risk for prostate cancer incidence was 1.32 (95% CI 0.80–2.18). More recent evidence reports no association between α-linolenic acid intake and risk of prostate cancer [49,50] (also, Simon JA, Tanzman JS, Sabaté J: Lack of effect of walnuts on serum levels of prostate specific antigen: a brief report, submitted). The Lyon Diet Heart Study, a randomized secondary prevention trial, found that subjects consuming a Mediterranean diet with 0.8% of calories from α-linolenic acid were not at increased risk of prostate cancer compared to those consuming a diet that met the criteria of an American Heart Association Step I Diet [49]. The prospective study conducted by Koralek et al [50] with 29,592 participants (mostly Caucasian) of the Prostate, Lung, Colorectal, and Ovarian (PLCO) Cancer Screening Trial reported no association between dietary intake of total α-linolenic acid (multivariate relative risk for highest vs. lowest quintile of intake/d [1.75 g/d vs. 1.38 g/d] was 0.94, 95% CI = 0.81–1.09) and α-linolenic acid from specific food sources and risk of total prostate cancer or prostate tumors that were defined by stage and grade. Consistent with our study results, Simon et al (Simon JA, Tanzman JS, Sabaté J: Lack of effect of walnuts on serum levels of prostate specific antigen: a brief report, submitted) reported that short term consumption of walnuts did not affect PSA levels adversely in healthy men.

## Conclusion

Our data offer no support that walnuts, which are high in α-linolenic acid, increase prostate cancer risk, as measured by serum PSA, androgen hormones and urinary symptoms, the primary markers of prostate health. In contrast, our results suggest that walnuts may be beneficial and improve serum γ-T and α-T: γ-T, two biomarkers that are important in prostate and vascular health. However, additional research is needed to assess the long-term efficacy of walnut consumption on PSA and other primary measures of prostate health.

## Abbreviations used

PSA: prostate specific antigen; PFP: percent free PSA; α-T: alpha-tocopherol; γ-T: gamma-tocopherol; IGF-1: insulin-like growth factor; CVD: cardiovascular disease; TG: triglycerides; NHANES: National Health And Nutrition Examination Survey.

## Competing interests

The authors declare that they have no competing interests.

## Authors' contributions

TH and PKE conceived of the study design and obtained funding. KS drafted the manuscript, assisted with sample collection and performed the statistical analyses with SW and FL. WS performed the red blood cell and lipid analyses. DB and VF served as project managers and directed the sample collection. All authors read and approved the final manuscript.
